# Supplementary material for: Empirical methods for controlling false positives and estimating confidence in ChIP-Seq peaks
Source: BMC Bioinformatics. 2008 Dec 5;9:523. doi: 10.1186/1471-2105-9-523 (PMC2628906; doi:10.1186/1471-2105-9-523)
Supplement: Additional File 1 — A variety of html documents from the USeq web site detailing the available applications, their best usage, output file type descriptions, command line menus, etc. [file 1471-2105-9-523-S1.zip › USeqUserGuides/JavaDoc.zip!/JavaDoc/bioroot/plasmid/PlasmidBean.html]

PlasmidBean


|  |  |  |
| --- | --- | --- |
| **Overview** \| **Package** \| **Class** \| **Use** \| **Tree** \| **Deprecated** \| **Index** \| **Help** | |  |
| **PREV CLASS**   **NEXT CLASS** | **FRAMES**    **NO FRAMES**     **All Classes** |  |
| SUMMARY: NESTED \| FIELD \| CONSTR \| METHOD | DETAIL: FIELD \| CONSTR \| METHOD |  |


---


## bioroot.plasmid Class PlasmidBean

```
java.lang.Object
  bioroot.ReagentBean
      bioroot.plasmid.PlasmidBean
```

**All Implemented Interfaces:**: java.lang.Comparable

---

``` public class PlasmidBean extends ReagentBean ```

**Author:**
:   nix
    Container for holding plasmid information

---

**Field Summary**

**Fields inherited from class bioroot.ReagentBean**

`log`


**Constructor Summary**

`PlasmidBean()`

`PlasmidBean(int id,
DBUtil bioRoot)`

`PlasmidBean(java.sql.ResultSet results)`

`PlasmidBean(java.lang.String[] cells,
IdNameMatch[] genes,
IdNameMatch[] organisms,
IdNameMatch[] markerIDMatch,
UserBean userBean,
DBUtil bioRoot)`
  
          For bulk file upload should be 16 cells
0 1 2 3 4 5 6 7 8 9 10 11 12 13 14 15 16
Name Alias Source Recipients Reference Gene Organism Markers StrainIds Location Concentration Volume Purification Availability VisibilityBean Notes Sequence


| **Method Summary** | |
| --- | --- |
| `int` | `compareTo(java.lang.Object obj)` |
| `java.lang.String` | `getConcentration()` |
| `java.lang.String` | `getFileName()` |
| `int` | `getGeneId()` |
| `java.lang.String` | `getGeneName(DBUtil bioRoot)` |
| `java.lang.String` | `getHotLinkedOligos()`             Returns HTML for of oligoMatches for display. |
| `java.lang.String` | `getHotLinkedStrains(DBUtil bioRoot)`             Returns HTML strain names for display. |
| `int` | `getLength()` |
| `java.lang.String[]` | `getMarkers(DBUtil bioRoot)` |
| `OligoMatch[]` | `getOligoMatches()` |
| `java.lang.String` | `getPlainTextOligos()`             Returns plain text of oligoMatches for archive. |
| `java.lang.String` | `getPlainTextStrain()`             Returns plain text of strain ids for archive. |
| `java.lang.String` | `getPurification()` |
| `java.lang.String` | `getRecipients()` |
| `java.lang.String` | `getRestrictionCutSites(Enzyme[] enzymes)` |
| `java.lang.String` | `getSequence()` |
| `java.lang.String` | `getSource()` |
| `java.lang.String[]` | `getStrainConcats(DBUtil bioRoot)`             Returns an array of id: strainName, or empty String[]{""} if there are none. |
| `java.lang.String` | `getVolume()` |
| `boolean` | `isBlasted()` |
| `boolean` | `isPlasmidNameUnique(DBUtil bioRoot)`             checks to see if plasmid name is unique within labgroup, assumes a connection has been made |
| `void` | `loadPlasmidBean(java.sql.ResultSet results)` |
| `boolean` | `saveMarkers(DBUtil bioRoot, boolean deleteOld)` |
| `boolean` | `saveStrainIds(DBUtil bioRoot, boolean deleteOld)`             Can delete old strainIds from PlasmidStrain table, and add new entries if any are present in the strainConcats. |
| `void` | `setBlasted(boolean blasted)` |
| `void` | `setConcentration(java.lang.String ngUl)` |
| `void` | `setFileName(java.lang.String fileName)` |
| `void` | `setGeneId(int i)` |
| `void` | `setGeneName(java.lang.String string)` |
| `void` | `setMarkers(java.lang.String[] markers)` |
| `void` | `setOligoMatches(OligoMatch[] oligoMatches)` |
| `void` | `setPurification(java.lang.String string)` |
| `void` | `setRecipients(java.lang.String string)` |
| `void` | `setSequence(java.lang.String string)` |
| `void` | `setSource(java.lang.String source)` |
| `void` | `setStrainConcats(java.lang.String[] strainConcats)` |
| `void` | `setVolume(java.lang.String d)` |
| `boolean` | `submitNew(DBUtil bioRoot)` |
| `boolean` | `updateOld(DBUtil bioRoot, UserBean submitter)` |

**Methods inherited from class bioroot.ReagentBean**

`appendMessages, canModifyAll, canModifyCommon, canView, getAlias, getAvailability, getComments, getEditHistory, getGenericVisibility, getId, getLabGroupId, getLabGroupName, getLastUser, getLocation, getMessages, getName, getNotes, getOrderBy, getOrganismId, getOrganismName, getOrganization, getOwnerId, getOwnerName, getReference, getVisibility, isComplete, isReagentNameUnique, loadReagentBean, setAlias, setAvailability, setComments, setComplete, setEditHistory, setId, setLabGroupId, setLabGroupName, setLastUser, setLocation, setMessages, setName, setNotes, setOrderBy, setOrganismId, setOrganismName, setOwnerId, setOwnerName, setReference, setVisibility, updateCommonAccessFields`

**Methods inherited from class java.lang.Object**

`equals, getClass, hashCode, notify, notifyAll, toString, wait, wait, wait`

**Constructor Detail**

### PlasmidBean

```
public PlasmidBean()
```

---


### PlasmidBean

```
public PlasmidBean(int id,
                   DBUtil bioRoot)
```

---


### PlasmidBean

```
public PlasmidBean(java.sql.ResultSet results)
```

---


### PlasmidBean

```
public PlasmidBean(java.lang.String[] cells,
                   IdNameMatch[] genes,
                   IdNameMatch[] organisms,
                   IdNameMatch[] markerIDMatch,
                   UserBean userBean,
                   DBUtil bioRoot)
```

:   For bulk file upload should be 16 cells
    0 1 2 3 4 5 6 7 8 9 10 11 12 13 14 15 16
    Name Alias Source Recipients Reference Gene Organism Markers StrainIds Location Concentration Volume Purification Availability VisibilityBean Notes Sequence


**Method Detail**

### compareTo

```
public int compareTo(java.lang.Object obj)
```

:   **Specified by:**: `compareTo` in interface `java.lang.Comparable` **Overrides:**: `compareTo` in class `ReagentBean`

---


### getLength

```
public int getLength()
```

---


### getRestrictionCutSites

```
public java.lang.String getRestrictionCutSites(Enzyme[] enzymes)
```

---


### getGeneName

```
public java.lang.String getGeneName(DBUtil bioRoot)
```

---


### getMarkers

```
public java.lang.String[] getMarkers(DBUtil bioRoot)
```

---


### setMarkers

```
public void setMarkers(java.lang.String[] markers)
```

---


### saveMarkers

```
public boolean saveMarkers(DBUtil bioRoot,
                           boolean deleteOld)
```

---


### getHotLinkedOligos

```
public java.lang.String getHotLinkedOligos()
```

:   Returns HTML for of oligoMatches for display.

---


### getPlainTextOligos

```
public java.lang.String getPlainTextOligos()
```

:   Returns plain text of oligoMatches for archive.

---


### setStrainConcats

```
public void setStrainConcats(java.lang.String[] strainConcats)
```

---


### getStrainConcats

```
public java.lang.String[] getStrainConcats(DBUtil bioRoot)
```

:   Returns an array of id: strainName, or empty String[]{""} if there are none.

---


### saveStrainIds

```
public boolean saveStrainIds(DBUtil bioRoot,
                             boolean deleteOld)
```

:   Can delete old strainIds from PlasmidStrain table, and add new entries if any are
    present in the strainConcats.

---


### getHotLinkedStrains

```
public java.lang.String getHotLinkedStrains(DBUtil bioRoot)
```

:   Returns HTML strain names for display.

---


### getPlainTextStrain

```
public java.lang.String getPlainTextStrain()
```

:   Returns plain text of strain ids for archive.

---


### loadPlasmidBean

```
public void loadPlasmidBean(java.sql.ResultSet results)
```

---


### submitNew

```
public boolean submitNew(DBUtil bioRoot)
```

---


### updateOld

```
public boolean updateOld(DBUtil bioRoot,
                         UserBean submitter)
```

---


### isPlasmidNameUnique

```
public boolean isPlasmidNameUnique(DBUtil bioRoot)
```

:   checks to see if plasmid name is unique within labgroup, assumes a connection has been made

---


### getPurification

```
public java.lang.String getPurification()
```

---


### getSequence

```
public java.lang.String getSequence()
```

---


### getVolume

```
public java.lang.String getVolume()
```

---


### setGeneName

```
public void setGeneName(java.lang.String string)
```

---


### setGeneId

```
public void setGeneId(int i)
```

---


### setPurification

```
public void setPurification(java.lang.String string)
```

---


### setSequence

```
public void setSequence(java.lang.String string)
```

---


### setVolume

```
public void setVolume(java.lang.String d)
```

---


### getGeneId

```
public int getGeneId()
```

---


### getFileName

```
public java.lang.String getFileName()
```

---


### setFileName

```
public void setFileName(java.lang.String fileName)
```

---


### getConcentration

```
public java.lang.String getConcentration()
```

---


### setConcentration

```
public void setConcentration(java.lang.String ngUl)
```

---


### getSource

```
public java.lang.String getSource()
```

---


### setSource

```
public void setSource(java.lang.String source)
```

---


### setOligoMatches

```
public void setOligoMatches(OligoMatch[] oligoMatches)
```

---


### getOligoMatches

```
public OligoMatch[] getOligoMatches()
```

---


### isBlasted

```
public boolean isBlasted()
```

---


### setBlasted

```
public void setBlasted(boolean blasted)
```

---


### getRecipients

```
public java.lang.String getRecipients()
```

---


### setRecipients

```
public void setRecipients(java.lang.String string)
```


---


|  |  |  |
| --- | --- | --- |
| **Overview** \| **Package** \| **Class** \| **Use** \| **Tree** \| **Deprecated** \| **Index** \| **Help** | |  |
| **PREV CLASS**   **NEXT CLASS** | **FRAMES**    **NO FRAMES**     **All Classes** |  |
| SUMMARY: NESTED \| FIELD \| CONSTR \| METHOD | DETAIL: FIELD \| CONSTR \| METHOD |  |


---
